# Relationship between radiocaesium in muscle and physicochemical fractions of radiocaesium in the stomach of wild boar

**DOI:** 10.1038/s41598-020-63507-5

**Published:** 2020-04-22

**Authors:** Rie Saito, Yui Nemoto, Hirofumi Tsukada

**Affiliations:** 1 Fukushima Prefectural Centre for Environmental Creation, 10-2 Fukasaku, Miharu-machi, Fukushima 963-7700 Japan; 2 Institute of Environmental Radioactivity, Fukushima University, 1 Kanayagawa, Fukushima-shi, Fukushima 960-1296 Japan

**Keywords:** Ecology, Ecology

## Abstract

After the accident at the TEPCO Fukushima Daiichi Nuclear Power Plant in 2011, it became important to study radiation dynamics, assess internal radiation exposure and specify factors affecting radionuclide variation in wildlife. Therefore, it is necessary to investigate which physicochemical fractions of radiocaesium (^137^Cs) are absorbed from ingested material in species with high activity concentrations of ^137^Cs, such as wild boar. This study analysed the physicochemical fractions of ^137^Cs in the stomach contents of wild boar to evaluate the transfer from ingested food to muscle. The ^137^Cs activity concentration in muscle showed a significantly positive relationship with the ^137^Cs activity concentration in the exchangeable fraction, and the sum of the ^137^Cs activity concentrations in the exchangeable and bound to organic matter fractions. Seasonal variations were also found in the ^137^Cs activity concentration in the exchangeable fraction, and the sum of the ^137^Cs activity concentrations in the exchangeable and bound to organic matter fractions. These findings suggest that the proportions of the physicochemical fractions of ^137^Cs in the exchangeable and bound to organic matter fractions in the stomach contents are important factors affecting the increases and seasonal dynamics of the activity concentrations of ^137^Cs in wild boar muscle.

## Introduction

In March 2011, large amounts of radionuclides were released into the environment as a result of an accident at Tokyo Electric Power Company’s Fukushima Daiichi Nuclear Power Plant (FDNPP). Approximately 13–15 PBq of caesium-137 (hereinafter, ^137^Cs, physical half-life of 30 years) was released from the FDNPP accident^1,2^, and as a result, ^137^Cs became a major source of radiation contaminating the environment. Since ^137^Cs has high bioavailability (i.e. absorption and transfer rates) due to having chemical characteristics similar to those of the monovalent cation potassium, there has been concern that ^137^Cs will accumulate in wildlife over the long-term^3^. Indeed, ^137^Cs has been detected in many wildlife species (e.g. insects^4^, frogs^5^, fishes^6–8^, birds^9^, mammals^10,11^) since the FDNPP accident.

After the FDNPP accident, serious radionuclide fallout occurred over extensive areas of north-eastern Japan. As most of this region is covered by forests (approximately 70%), the long-term ^137^Cs contamination of forestry ecosystems has been a concern^12^. Due to its long-term retention (ca. 10–100 years) at high concentrations, radiocaesium that has been deposited in forests is considered to have a long ecological half-life^13,14^. The dynamics of ^137^Cs in forestry ecosystems can be roughly divided into two stages: the “early” stage, in which the distribution of radiocaesium within the system occurs promptly between the soil and trees, and the “steady state” stage, in which there are long-term changes in the subsequent distribution of radiocaesium among organisms^14,15^. Generally, immediately after deposition in the soil following release into the atmosphere by an accident, a high proportion of the radiocaesium released exists in an ionic state, which is easily absorbed by plants. However, after physical and chemical changes in the soil following deposition, radiocaesium binds to soil particles over time and becomes less easily dissolvable^16^. Therefore, radiocaesium in soil can generally be separated into: (1) an exchangeable fraction; (2) a bound to organic matter fraction; and (3) and a particle-bound fraction (also called a “strongly bound fraction”). Radiocaesium in the exchangeable fraction is substitutable with monovalent cations, which have an ionic radius similar to that of caesium and are absorbed by negatively charged sites in organic matter and soil particles. Radiocaesium in the bound to organic matter fraction is bound to organic matter, whereas radiocaesium in the strongly bound fraction is a specific fraction for caesium strongly bound to clay mineral layers, and is difficult to elute^17^. Thus, radiocaesium exists as different types, or physicochemical fractions, in the environment. These physicochemical fractions also change gradually over time in the environment, from fractions in which radiocaesium can be transferred relatively easily to those in which it is relatively difficult to transfer; for example, the rate of radiocaesium transfer to plants decreases over time^18^. These changes over time in the physicochemical fractions of radiocaesium are considered to influence the rates of transfer of radioactive nuclides to wildlife. Consequently, to clarify radiocaesium transfer from the environment to organisms, both the concentration and physicochemical fractions of radiocaesium in the environment need to be determined.

In this study, we examined the relationship between the physicochemical fractions of ^137^Cs in the environment and the bioavailability of ^137^Cs in wild boars (*Sus scrofa*), which mainly inhabit the forests and countryside surrounding the FDNPP, and for which investigational data on the transfer of ^137^Cs in the environment have been accumulated. Monitoring results in Fukushima Prefecture after the FDNPP accident showed that wild boar tended to have particularly higher activity concentrations of ^137^Cs in muscle tissue than did other wildlife species^10,19^. Studies have also shown that wild boar captured in areas with similar levels of soil contamination exhibit extremely large inter-individual variations in the activity concentrations of ^137^Cs^19^. Studies conducted after the Chernobyl Nuclear Power Plant accident also reported that wild boar had high activity concentrations of ^137^Cs, which enters the muscles after the digestion of food, and that the accumulation of ^137^Cs occurred over extended periods^20–23^. Regarding the activity concentration of radiocaesium in wild boar after the FDNPP accident, a monitoring survey conducted by the Fukushima Prefectural Government in 2017 detected animals in which the fresh mass (hereinafter, “FM”) activity concentration of radiocaesium was several thousand to a ten thousand Bq kg^–1^ at more than 6 years after the accident^24^. In addition, seasonal changes have been reported in the activity concentration of ^137^Cs in wild boar muscle;^19,25^ however, the factors that affected these fluctuations in wild boar after the FDNPP accident remain unclear.

Internal exposure to anthropogenic radioactive materials in animals is typically via the ingestion of radiation-contaminated food^23,26^. Our previous study found a significant positive relationship between the activity concentration of ^137^Cs in the muscle and stomach contents of wild boars, clearly indicating that ingested material influences the activity concentration of ^137^Cs in wild boar muscle. Wild boars are omnivores that feed mainly on plants (e.g. leaves, roots, subterranean stems), as well as earthworms, insects and other small animals^27^. It is likely that wild boars also passively ingest soil while eating these foods. While the activity concentration of ^137^Cs in soil is typically several orders of magnitude higher than that in plants, the elutability of ^137^Cs in water is very limited because much of the ^137^Cs that exists in soil is in the strongly bound fraction. In wild animals, it is unlikely that all of the ^137^Cs contained in the orally ingested material is absorbed; rather, only the ^137^Cs that is eluted from the ingested material is absorbed and distributed in the body. In terms of studying radiation dynamics, assessing internal radiation exposure and identifying the factors responsible for variable levels of radionuclides in wildlife, it is important to study wildlife species with high ^137^Cs levels, such as wild boar; specifically, it is important to clarify the fractions of ^137^Cs that are absorbed from the ingested material and the degree of their influence on the activity concentration of ^137^Cs in the body. Therefore, this study aimed to analyse the physicochemical fractions of ^137^Cs contained in the diet of wild boars by examining their stomach contents and clarifying the physicochemical fractions of ^137^Cs that could be eluted from these contents.

Wild boar utilize most of their annual home range in a day^28,29^, and utilize the same area every day^28^. Consequently, their stomach contents reflect individual food habits over a relatively long period (Nemoto *et al*., in prep.). Stomach contents are thus a valuable tool for evaluating the food habits of wild boar. For these reasons, we examined the relationships between the concentration of ^137^Cs physicochemical fractions in the stomach contents and seasonal variations in the activity concentrations of ^137^Cs in muscle.

## Methods

### Samples

**Figure 1:**
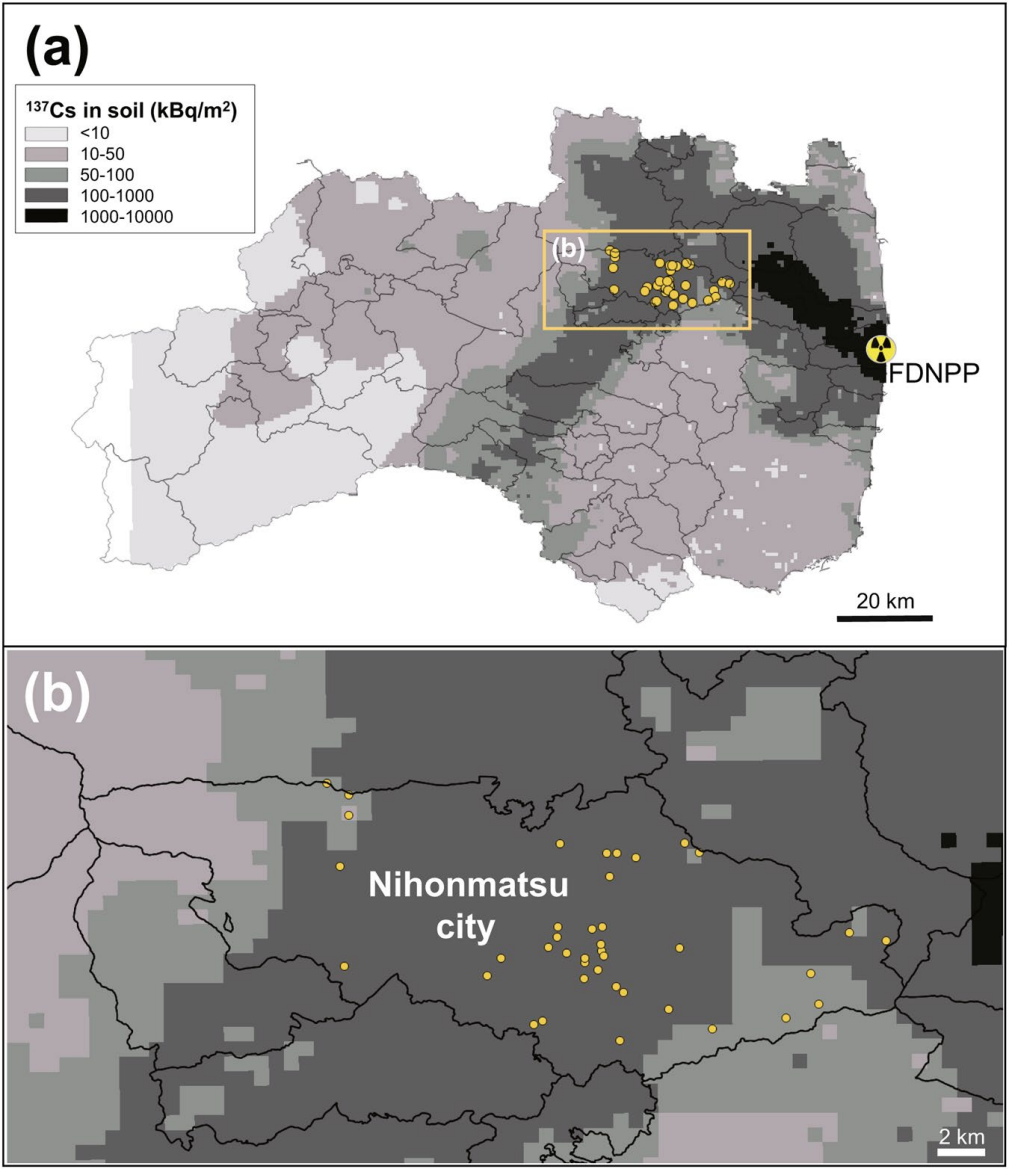
Study area and locations where the wild boars were captured. Orange dots indicate the points at which the wild boars were captured. Data on the amount of radiocaesium (^137^Cs) deposits show the investigation results obtained by the Japan Atomic Energy Agency’s (JAEA) 5th airborne monitoring survey (https://emdb.jaea.go.jp/emdb/en/portals/b1020201/). (**a**) The entire area of Fukushima Prefecture and (**b**) Nihonmatsu city. This figure was created using QGIS 2.1.8 (https://www.qgis.org/en/site/). The map of Fukushima prefecture was obtained by Ministry of Land, Infrastructure, Transport and Tourism (MLIT) of Japan (http://nlftp.mlit.go.jp/ksj/).

We used samples from 40 wild boars (males: 24, females: 16) captured and killed by hunters in Nihonmatsu city, Fukushima Prefecture, from July to October in 2015 (Fig. 1). The number of samples of each sex (male and females, respectively) captured in each month is as follows: July: 4 and 3, August: 10 and 2, September: 6 and 6, and October: 4 and 5. The wild boars were captured by hunters as part of harmful wildlife control efforts implemented under the Wildlife Protection and Hunting Management Law (Law No. 32, 1918). Therefore, wild boars were not killed specifically for this research and no live animals were used. The wild boars were captured using a binding trap, which does not require any bait. Muscle and stomach samples were harvested from each dead wild boar. The muscle samples were minced after removing tendons and fat as much as possible, and then encapsulated in U8 containers (100 ml, φ56 mm × 68 mm). The stomach contents were stirred well and also encapsulated in U8 containers. To analyse the physicochemical fractions, a portion of the stomach contents was freeze-dried for several days, followed by crushing and mixing using a cutter blender. We followed all guidelines for the ethical treatment of animals in research by The Mammal Society of Japan^30^.

### Measurements

Gamma ray-emitting radionuclides in the samples encapsulated in the U8 containers were measured using a germanium semiconductor detector (Canberra GC2020, GC3020 and GC4020; Mirion Technologies (Canberra) KK, Tokyo, Japan) connected to a multichannel analyser.

### Analysis of physicochemical fractions

The physicochemical fractions of ^137^Cs in the stomach contents were investigated by classification into the following three fractions based on the physicochemical fractions of ^137^Cs existing in the soil: (1) an exchangeable fraction; (2) a bound to organic matter fraction; and (3) a strongly bound fraction. We assumed that (1) the exchangeable fraction is bound relatively loosely and is absorbed in the gastrointestinal tract; (2) the bound to organic matter fraction is bound to organic matter and is partly absorbed during the course of digestion; and (3) the strongly bound fraction is excreted without being absorbed into the body.

Each physicochemical fraction was extracted using the following methods.

1. Exchangeable fraction
  One gram of dried sample was added to 10 mL of 1 M ammonium nitrate solution (with a solid–liquid ratio of 1:10) and stirred for 1 hour, followed by filtration using a 0.22 μm membrane filter (MilliporeSigma, Burlington, MA, USA) The filtrate containing only the ^137^Cs exchangeable fraction was then retrieved and encapsulated in a U8 container.
2. Bound to organic matter fraction
  One gram of dried sample was added to hydrogen peroxide solution and organic matter was decomposed at 80 °C. After adjusting the solid–liquid ratio to 1:7.5, 2.5 mL of 3.2 M ammonium acetate solution (20% nitric acid) was added to extract the ^137^Cs (with a solid–liquid ratio of 1:10). The residues were then added and rinsed in the same amount (10 mL) of purified water, followed by mixing with the extraction liquid. Because the filtrate contained the exchangeable and bound to organic matter fractions, the activity concentration of the ^137^Cs in the bound to organic matter fraction was calculated by subtracting the concentration of ^137^Cs in the exchangeable fraction (obtained in (1)) from that of ^137^Cs in the filtrate.
3. Strongly bound fraction

The activity concentration of the ^137^Cs in the strongly-bound fraction was calculated by adding the activity concentration of ^137^Cs in the exchangeable fraction in (1) and that in the bound to organic matter fraction in (2) above, and then subtracting this total from the activity concentration of ^137^Cs obtained for the dried stomach content samples.

### Statistical analysis

**Figure 2:**
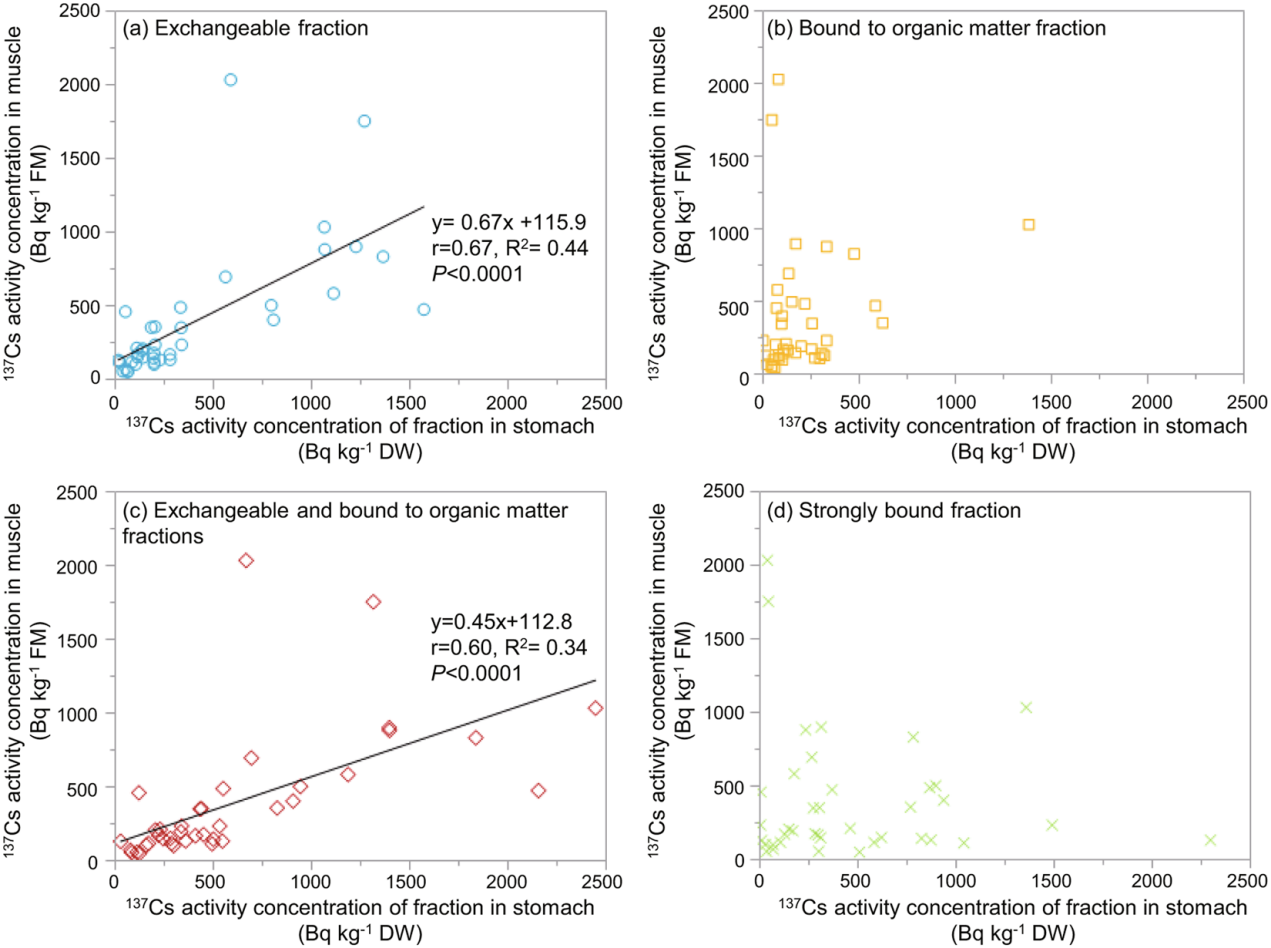
Relationship between the activity concentration of radiocaesium (^137^Cs) in wild boar muscle tissue and in different fractions of stomach contents. The single regression line equation, adjusted R-square (R^2^) and correlation coefficient (r) are shown only for the cases where a significant difference was observed between the two variables by regression and correlation analyses. DW: Dry weight, FM: Fresh mass.

**Figure 3:**
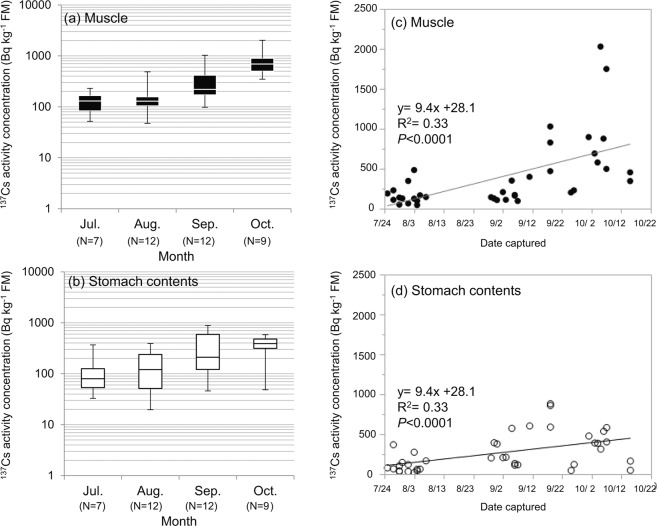
Seasonal variations in the activity concentration of radiocaesium (^137^Cs) in wild boar muscle tissue and stomach contents. (**a**,**b**) Monthly changes in the activity concentration of ^137^Cs in muscle and stomach contents from July to October in 2015. The upper and lower ends of the box plot indicate the 75th and 25th percentiles, respectively. The horizontal bar in the plot indicates the median value. The whiskers at the upper and lower ends of the box plot indicate the maximum and minimum values, respectively. (**c**,**d**) Changes in the activity concentration of ^137^Cs in muscle and stomach contents on the dates when the animals were captured. The single regression line equation and adjusted R-square (R^2^) are shown only for the cases where a significant difference was observed between the two variables in regression analysis. FM: Fresh mass.

**Figure 4:**
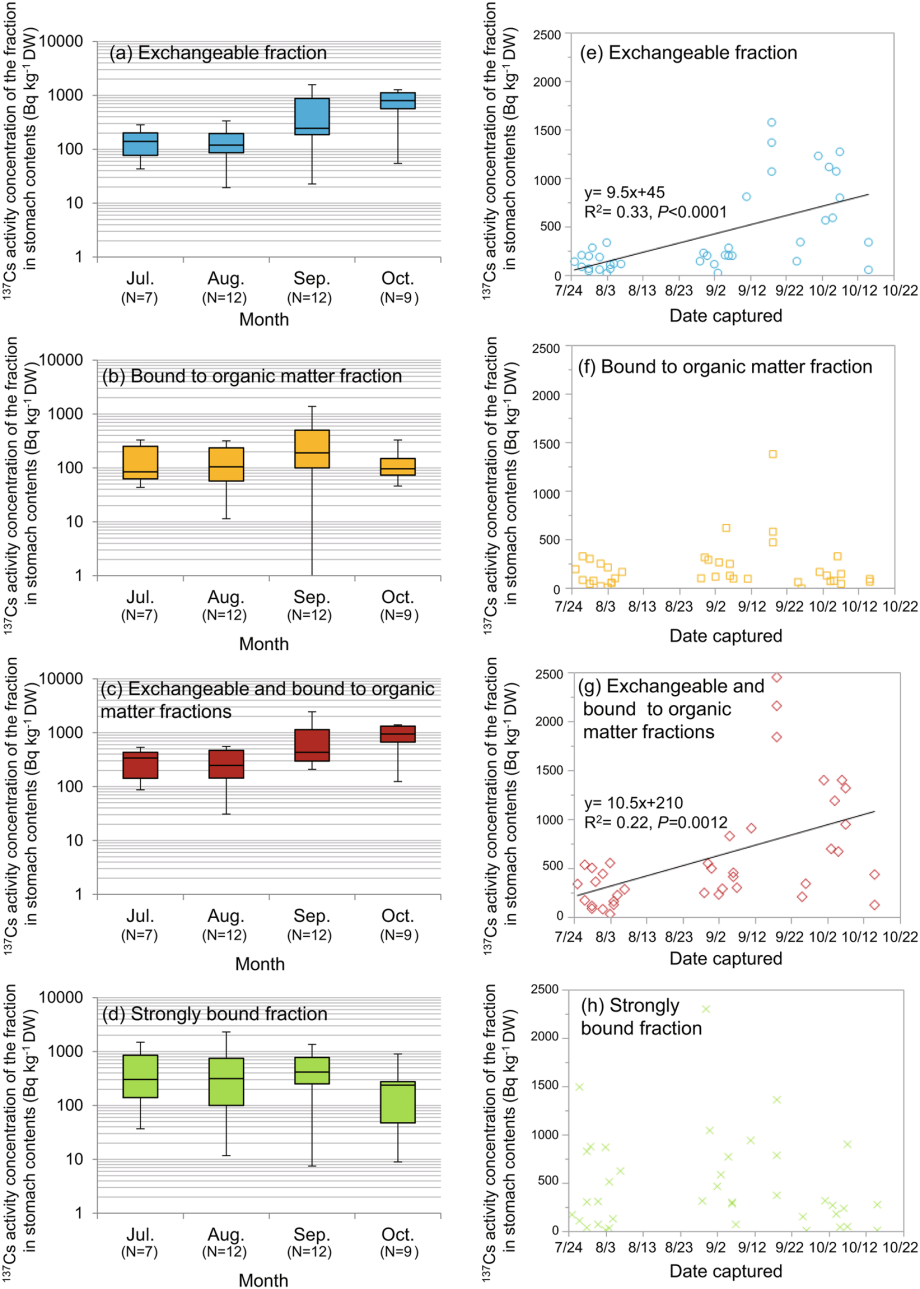
Seasonal variations in the activity concentrations of radiocaesium (^137^Cs) in different fractions of stomach contents. (**a**–**d**) Monthly changes in the activity concentrations of ^137^Cs in different fractions from July to October in 2015. The upper and lower ends of the box plot indicate the 75th and 25th percentiles, respectively. The horizontal bar in the plot indicates the median value. The whiskers at the upper and lower ends of the box plot indicate the maximum and minimum values, respectively. (**e**–**h**) Changes in the activity concentration of ^137^Cs in different fractions against dates on which animals were captured. The quadratic regression equation and adjusted R-square (R^2^) are shown only for the cases where a significant difference was observed between the two variables in regression analysis. DW: Dry weight.

We performed all statistical analyses using JMP 13.2.1 software (SAS, Cary, NC, USA). The relationships between the activity concentrations of ^137^Cs in muscle and in each physicochemical fraction of the stomach contents (Fig. 2), the date of capture and the activity concentration of ^137^Cs in muscle or stomach contents (Fig. 3) and date of capture and the activity concentrations of ^137^Cs of each physicochemical fraction (Fig. 3) were also analysed. In addition, because part or all of the bound to organic matter fraction was absorbed during digestion, we also analysed the sum of the exchangeable and bound to organic matter fractions (Figs. 2 and 4)

## Results

### Relationship between the activity concentration of ^137^Cs of each physicochemical fraction in the stomach contents and that of ^137^Cs in muscle

The activity concentrations of ^137^Cs in the muscle and stomach contents of wild boar samples examined in this study were in the ranges of 47–2030 and 20–833 Bq kg^–1^ FM, respectively, which represented a one- to two-order of magnitude difference, despite the fact that these samples were collected within a relatively limited geographical area (Nihonmatsu city, 344 km^2^). The percentages of the different physicochemical fractions of ^137^Cs in the stomach contents were 3–98%, 0–52% and 2–81% for the exchangeable, bound to organic matter and strongly-bound fractions, respectively, indicating marked differences among the boars. The mean proportion of the exchangeable fraction was 38 ± 24 (standard deviation [SD]) %, suggesting that approximately 40% of the ingested material was absorbed relatively promptly.

Figure 2 shows the relationship between the activity concentration of ^137^Cs in muscle and that in the physicochemical fractions of the stomach contents. The activity concentration of ^137^Cs in muscle showed a strong correlation with those in the following fractions of the stomach contents: (1) the activity concentration of ^137^Cs in the exchangeable fraction (in Bq kg^−1^ dry weight [hereinafter, “DW”]; r = 0.67, *P* < 0.0001, R^2^ = 0.44); and ((2) the activity concentration of ^137^Cs and the sum of ^137^Cs in the exchangeable and bound to organic matter fractions (in Bq kg^−1^ DW, r = 0.60, *P* < 0.0001, R^2^ = 0.34) (Fig. 2). In particular, the correlation coefficient was highest for the ^137^Cs in the exchangeable fraction.

### Seasonal variations in the physicochemical fractions of ^137^Cs

Figure 3 shows changes in the activity concentration of ^137^Cs in muscle and that in the stomach contents from July to October. The activity concentrations of ^137^Cs in muscle were similar in July and August and tended to increase in September and October. The activity concentration of ^137^Cs in the stomach contents showed a tendency to increase from July to October (Fig. 3). Seasonal variations were observed in the activity concentration of ^137^Cs in the muscle tissue of wild boars in Fukushima, and the activity concentration of ^137^Cs decreased from April to August, increased from September to November and remained high from December to March;^19^ the trend in the activity concentration of ^137^Cs in muscle in this study was roughly consistent with seasonal variations. Figure 4 shows changes in the activity concentrations of ^137^Cs in the physicochemical fractions of the stomach contents. Seasonal variations similar to those found in the activity concentration of ^137^Cs in muscle were observed in the activity concentrations of ^137^Cs of the exchangeable fraction and in the sum of the activity concentrations of ^137^Cs in the exchangeable and bound to organic matter fractions (Fig. 4). No significant relationship was found between the activity concentration of ^137^Cs in the bound to organic matter and strongly bound fractions (Fig. 4).

## Discussion

The results of the present study indicated that the activity concentration of ^137^Cs in muscle showed a strong correlation with that in (1) the activity concentration of ^137^Cs in the exchangeable fraction, and (2) the activity concentration of ^137^Cs of the sum of ^137^Cs in the exchangeable and bound to organic matter fractions. This suggests that the activity concentration of ^137^Cs in the exchangeable fraction of the ingested material, as well as that in the bound to organic matter fraction, all or part of which is considered to be digested in the stomach, are strongly related to the transfer of ^137^Cs to the body of a wild boar. No positive correlation was observed between the activity concentration of ^137^Cs in the strongly bound fraction and that in muscle, suggesting that the activity concentration of ^137^Cs in the strongly bound fraction did not have a marked influence on the accumulation of ^137^Cs in the body. In addition, ^137^Cs activity concentration levels were reported to be higher in the head and viscera than in the muscle of the freshwater fish ayu (*Plecoglossus altivelis*) in Fukushima^8^. This was thought to be because the viscera of these fish absorbs difficult to absorb particles (i.e. ^137^Cs in suspended fractions, such as silt and particulates) when consuming periphytic algae. In addition, part of the radiocaesium in the rectal contents is not absorbed by the body of wild boar, but rather, passes through in the faeces^25^. It has also been suggested that ingested material contains some ^137^Cs that is not taken into the body, and that most of the ^137^Cs in the strongly bound fraction existing in the stomach contents is excreted from instead of being absorbed by the body^25^. If the physicochemical fractions of ^137^Cs in faeces excreted from the body are exchangeable or bound to organic matter fractions, then there is a risk that radionuclides may be further concentrated in dung beetles, fungi and other organisms that utilize the faeces of wild boars^25^. The results of this study showed that the percentage of the strongly bound fraction of ^137^Cs in the stomach contents was 42 ± 23%, ranging from 2–81% in all samples (Table 1). Since the ^137^Cs in the strongly bound fraction, which was not digested in the stomach, is likely to be concentrated and excreted in the faeces, it is expected that a substantial amount of ^137^Cs in the strongly bound fraction exists in faeces. When considering the influence of ^137^Cs on the ecosystem or the dynamics of ^137^Cs in the environment, it is important to monitor which physicochemical fractions of ^137^Cs are contained in faeces.

**Table 1:** Percentage of ^137^Cs physicochemical fraction in stomach contents.

	Rate of ^137^Cs physicochemical fraction in stomach contents (%)		
	Average ± SD	Maximum	Minimum
^137^Cs exchangeable fraction	38 ± 24	98	3
^137^Cs bound to organic matter fraction	20 ± 12	52	0
Sum of ^137^Cs exchangeable and ^137^Cs bound to organic matter fractions	58 ± 23	98	19
^137^Cs strongly bound fraction	42 ± 23	81	2

In addition, no significant relationship was found between the activity concentration of ^137^Cs in the bound to organic matter and strongly bound fractions, which suggests that the concentrations of the exchangeable and bound to organic matter fractions that may be eventually liquated are both important factors affecting the seasonal variation in the activity concentrations of ^137^Cs in muscle. However, the extent to which the bound to organic matter fraction is liquated and absorbed remains unclear. To elucidate the contribution of the bound to organic matter fraction in the stomach contents to the activity concentrations of ^137^Cs in the body, it will be necessary to clarify the extent to which the bound to organic matter fraction is liquated in the stomach. A previous investigation of wild boars reported that the stomach contents contained fibres derived mainly from bamboo in May and June, dicotyledonous plants from July to September and tubers from October to March^31^. That study also identified seasonal changes in the plant parts and materials ingested by wild boars, which implies that the amount of soil ingested is expected to increase when the wild boars feed primarily on tubers. It therefore seems likely that these seasonal changes in food habits may have a significant impact on the physicochemical fractions of ^137^Cs absorbed by wild boar. In future research, we intend to examine the seasonal food habits of wild boars to elucidate the relationships between, and changes in, the physicochemical fractions of ^137^Cs contained in ingested materials and the absorption of ^137^Cs by the body.
